# Strategies and Tools for Electronic Health Records and Physician Workflow Alignment: Protocol for a Scoping Review

**DOI:** 10.2196/60464

**Published:** 2025-06-02

**Authors:** Oluwakemi Olufunmilayo Oluwole, Nicole Haggerty, Uche Ikenyei, Oluwabambi Tinuoye, Andreawan Honora, Mohammed Abass Issakah

**Affiliations:** 1 Health Information Science Department Faculty of Information and Media Studies University of Western Ontario London, ON Canada; 2 Information Systems Ivey Business School University of Western Ontario London, ON Canada

**Keywords:** electronic health records, electronic medical records, physician workflow, alignment, optimization, scoping review, JBI framework, workflow, implementation, PRISMA

## Abstract

**Background:**

Electronic health records (EHRs) have been widely adopted in health care systems globally, offering potential benefits in data accessibility, quality improvement, and enhanced patient outcomes. However, the alignment between EHRs and physician workflows remains a significant challenge, leading to negative impacts on physician well-being and patient care. While health care organizations have attempted various strategies to improve this alignment, critical gaps still persist, highlighting the need for a comprehensive understanding of existing approaches and their effectiveness as a means to chart effective strategies to align physician workflows with EHRs.

**Objective:**

This scoping review aims to identify and synthesize the strategies and tools health care organizations have used to align physician workflows with EHRs. This review will provide a toolkit for health care organizations and researchers, offering insights into effective alignment practices and identifying knowledge gaps for future research.

**Methods:**

This scoping review will follow the Joanna Briggs Institute framework for scoping reviews while incorporating the PRISMA-ScR (Preferred Reporting Items for Systematic Reviews and Meta-Analyses extension for Scoping Reviews) checklist for transparent reporting. We will search multiple databases, including MEDLINE, PubMed, Cochrane, CINAHL, Scopus, Embase, and Web of Science, for relevant literature on tools, strategies, and interventions used to align physician workflows with EHRs. The review will focus on studies involving physicians in direct patient care across primary, secondary, tertiary, and quaternary care settings. Two independent reviewers will screen titles, abstracts, and full texts for inclusion. Data extraction will be performed using a standardized form, and findings will be narratively synthesized and presented in tables and charts.

**Results:**

The study is expected to provide a comprehensive toolkit of strategies, tools, and interventions for EHR-physician workflow alignment. This synthesis will offer health care organizations practical guidance for improving alignment and provide researchers with a foundation for identifying research gaps and future directions. The final report is planned for submission to an indexed journal in August 2025.

**Conclusions:**

This scoping review will offer valuable insights into the strategies and tools implemented by health care organizations to align EHRs with physician workflows. By assessing the effectiveness and limitations of these approaches, the review aims to contribute to improved EHR usability, reduced physician burnout, and enhanced patient care.

**International Registered Report Identifier (IRRID):**

PRR1-10.2196/60464

## Introduction

The adoption of electronic health records (EHRs) is increasing in national and international health care systems [1]. EHRs are rapidly becoming the preferred method of digitizing health data, making it more accessible to patients and improving quality and analytical opportunities that enhance patient outcomes [2,3]. However, despite the recognized potential of EHRs to improve health care, the claim that they enhance workflow efficiency and productivity appears to be exaggerated [4,5]. The complex and emergent nature of health care, variations in individual physicians’ treatment processes, as well as varying needs of patients sometimes conflict with standardized processes in EHRs, leading to workflow challenges [6].

Workflow consists of activities organized in sequential processes, including the necessary resources to complete the task [5]. It is a series of linked steps based on the flow paradigm, distinct from a single task. Workflow is a model that represents the steps involved in a real work environment to move a work description from initiation to completion. For instance, the steps involved in patient-physician interaction, from a patient’s arrival at the physician’s office to their departure, can be depicted as a workflow [6]. However, while the duties of some health professionals, such as nurses, tend to follow relatively standardized routines, the tasks, task sequences, and priorities of physicians vary greatly by individual style and patient needs [7]. Also, physician workflows, especially in time-bound units such as the emergency and intensive care units, are nonlinear and complex. They depend on a wide variety of data inputs and may involve multitasking and fragmentation [8]. Physician workflows also include a wide range of data management and multilevel communication, such as physician-to-physician, physician-to-nurse, and physician-to-patient [9]. Consequently, it can be challenging to use a standardized EHR workflow specifically designed for clinicians to other interaction groups.

Several issues leading to misalignment between EHR and physician workflow have been documented in the EHR literature [5,10,11]. For instance, some authors have noted that EHR designers who are not health care professionals may not realize the importance of making EHRs adaptable, flexible, and responsive to the unpredictable nature of physicians’ workflow [6,12]. These designers, in their pursuit to enhance the value of EHRs, may inadvertently hinder physicians’ clinical routines and workflow, resulting in usability issues. Moreover, it can be argued that EHRs are not designed to automate physicians’ existing paper-based workflows [6].

The negative impact of workflow interference on physicians is significant and has been identified as a major contributor to burnout [10,11,13]. Furthermore, inadequate physician workflow design in the EHR can impede effective physician-patient interaction, restrict assessment and treatment, and increase the risk of medication errors [5,14]. Suboptimal EHR-mediated workflows often lead to workarounds, which involve using alternative approaches instead of the recommended methods after system implementation [15]. Given that these challenges have a significant impact on physicians, leading to burnout and stress [16], they can result in incomplete and inaccurate patient documentation [5]. Considering that physician workflow is a crucial factor in maximizing the benefits of the EHR [17], it is essential to understand how to navigate the challenges around the alignment between EHR and physician workflow for successful EHR implementation, usage, and value recommendations. It is also important to understand the impact of existing strategies and tools on EHR and physician workflow alignment. An example of such a tool is the integrated software such as Dragon Naturally Speaking app which is a speech recognition software. This software often serves both as a voice-to-text tool and an EHR inbox management system [18,19]. It improves communication and minimizes the time spent on documentation. Another example of a strategy is order-sets optimization within EHRs bundle frequently required tests for specific illnesses or conditions [20,21], which reduces the time spent on orders and improves physician workflow.

Despite the increasing number of studies on how to improve alignment between clinicians and EHR workflows, the existing knowledge is highly diverse and fragmented, creating gaps in our comprehensive understanding. Different studies focus on various aspects of EHR-physician workflow alignment, such as time management, stress levels, and patient outcomes. For example, some studies analyze the amount of time physicians dedicate to EHR tasks compared with patient interaction, while others assess the influence of EHR on physician productivity and job satisfaction [22,23]. The studies in this field lack synthesis, as researchers often work in isolation instead of building upon each other’s findings. Consequently, the body of knowledge remains fragmented, without a clear and unified direction for improvement. One of the purposes of a scoping review is to improve understanding of complex issues that affect health care workers and EHRs and to map existing tools to improve this challenge [24,25]. We argue that, to advance understanding of how EHR-physician workflow alignment should be approached, it is necessary to map the intellectual structure of current knowledge through a scoping review. This review will synthesize the concepts and interventions from published articles that focus on EHR-physician workflow alignment, ultimately creating a comprehensive toolkit of knowledge and strategies to achieve maximum alignment and minimize obstacles in EHR implementations. This understanding can assist health care organizations in optimizing alignment, leading to improved usability, increased adoption, reduced medication errors, and decreased physician burnout [20,26]. In addition, it can provide guidance to software developers and vendors on users’ and health care organization needs and preferences on how to effectively align EHR and physician workflow. Therefore, a scoping review method was chosen to synthesize diverse evidence from published and gray literature [27].

## Methods

To ensure consistency in the conduct and reporting of scoping reviews, as well as maximize rigor, this study will follow the Joanna Briggs Institute (JBI) guideline for Scoping Reviews [27]. In addition, it will incorporate the recent methodological development on scoping reviews, the PRISMA-ScR (Preferred Reporting Items for Systematic Reviews and Meta-Analyses extension for Scoping Reviews) checklist into the main framework [28] (Multimedia Appendix 1).

### Consultation

Consulting stakeholders is a vital aspect of the scoping review method. In this study, a librarian from the Weldon Library at the University of Western Ontario helped develop the search strategy. Once the study is completed, we will seek feedback from physicians who frequently use the EHR in their workflow to ensure an accurate and comprehensive interpretation of the findings. Their objective perspective will help validate the results, identify biases, and enhance the credibility and rigor of the research.

### Step 1: Formulate a Research Question

The population, context, and concept (PCC) framework will be used to identify the main concepts of primary review questions and inform the development of search strategies [20] (Textbox 1). Using the PCC framework, our review will focus on the strategies and tools that health care organizations use to align physician workflow with EHR (concept) for physicians that have face-to-face interaction with their patients (population) at any level of care (context).

**Review questions.**

- What strategies and tools have health care organizations used to align physician workflows with electronic health records systems?
- How have these strategies and tools impacted the alignment between physician workflows and electronic health records, health care delivery, and patient care?
- What are the strengths and limitations of these strategies and tools?
- What are the gaps that currently exist in literature and what opportunities for future studies exist?

### Step 2: Identifying Studies Relevant to the Research Question

To answer the research questions, we will conduct a comprehensive search of several databases, including Embase (OVID), MEDLINE (OVID), PubMed, COCHRANE CINAHL, and interdisciplinary databases such as WEB OF SCIENCE and SCOPUS. To ensure we capture all relevant articles, we will also screen the references of selected articles using the ancestry method. We aim to include scientific peer-reviewed studies and conference proceedings, encompassing all types of scientific study designs and approaches. Furthermore, we will conduct a thorough search for gray literature by identifying pertinent sources such as reports, conference proceedings, theses, and government documents. We will search databases such as ProQuest Dissertations and Theses and OpenGrey to access and hand-pick studies that have not undergone peer review. Also, we will search conference proceedings for abstracts and presentations that may not have been published in journals and take the time to explore the websites of health care organizations and government agencies.

By using this strategy, a preliminary search of the PubMed database in December 2024 returned 516 articles. The sample search strategy is presented in Table 1. This strategy will be repeated for all the identified databases.

**Table 1:** A sample of the search query.

Search number	Search query	Items found
#1	Search: (((((Electronic Health Records[MeSH Terms]) OR (Electronic Medical records[MeSH Terms])) OR (EHR[Title/Abstract])) OR (EMR[Title/Abstract])) OR (Computerized Provider Order Entry[Title/Abstract])) OR (Electronic health records[Title/Abstract])	56,275
#2	Search: ((Physician workflow[Title/Abstract]) OR (Doctor workflow[Title/Abstract])) OR (Clinician workflow[Title/Abstract]) “physician workflow”[Title/Abstract] OR ((“doctor s”[All Fields] OR “doctoral”[All Fields] OR “doctorally”[All Fields] OR “doctorate”[All Fields] OR “doctorates”[All Fields] OR “doctoring”[All Fields] OR “physicians”[MeSH Terms] OR “physicians”[All Fields] OR “doctor”[All Fields] OR “doctors”[All Fields]) AND “workflow”[Title/Abstract]) OR “clinician workflow”[Title/Abstract]	2860
#3	#1 AND #2 Search: (#1) AND (#2) (“physician workflow”[Title/Abstract] OR ((“doctor s”[All Fields] OR “doctoral”[All Fields] OR “doctorally”[All Fields] OR “doctorate”[All Fields] OR “doctorates”[All Fields] OR “doctoring”[All Fields] OR “physicians”[MeSH Terms] OR “physicians”[All Fields] OR “doctor”[All Fields] OR “doctors”[All Fields]) AND “workflow”[Title/Abstract]) OR “clinician workflow”[Title/Abstract]) AND (“electronic health records”[MeSH Terms] OR “electronic health records“[MeSH Terms] OR “EHR”[Title/Abstract] OR “EMR”[Title/Abstract] OR “computerized provider order entry”[Title/Abstract] OR “electronic health records”[Title/Abstract])	516

### Step 3: Study Selection

A scoping review protocol should clearly outline the eligibility criteria (inclusion) and sources of information that will be used in the study [28]. The inclusion and exclusion criteria for selecting studies will be achieved based on the scope of the inquiry. Also, the ancestry approach will be adopted to identify relevant studies from the references of selected articles.

#### Inclusion Criteria (PCC)

The inclusion criteria for the studies will align with the PCC guidelines, which specify the PCC [29].

#### Population

The study will specifically target physicians who directly interact with patients, excluding non–patient-engaging physicians such as pathologists and radiologists. This inclusion is important because non–patient-engaging physicians interact with the EHR differently than physicians who typically combine documentation and review during patient interactions. Physicians who simultaneously interact with both the EHR and patients have been reported to experience issues such as divided attention and higher cognitive workload leading to burnout and stress [10,11,16]. The study will encompass articles focusing on physicians who use the EHR in primary, secondary, tertiary, and quaternary care (Textbox 2).

**Inclusion and exclusion criteria.**

**Inclusion criteria**

- Studies on electronic health records or electronic medical records
- Studies in the English language
- All publications that seek to optimize the usability of electronic health records for physician’s use by improving workflow
- Focuses on physician workflow
- Studies carried out at any level of care, primary, secondary, tertiary, and quaternary care
- Studies that focus on physicians who have direct contact with patients such as primary care physicians, internists, and surgeons
- Studies based on the workflow of a single physician or multiple physicians

**Exclusion criteria**

- Studies that focus on other health information systems excluding electronic health records and electronic medical records
- Studies not published in the English language
- Studies that do not address the alignment of electronic health records and physician workflow
- Focuses on other health care professionals such as nurses and caregivers
- Studies that focus on the impact of electronic health records on workflow or understanding of physician workflow
- Physician that does not have direct contact with patients
- Studies that target the concept of meaningful use will be excluded

The study will center on physicians, aiming to systematically document and describe the tools and strategies used to optimize their workflow with the EHR. While some studies on clinical workflow will be included in the search using the term “clinical workflow,” this is to ensure that studies that explore the combination of physician workflow with other clinician’s workflow are not overlooked. Consequently, we will exclude studies that solely concentrate on other clinicians, such as nurses and allied health professionals, during the screening process.

#### Concept

Our focus is to examine interventions, defined as practical actions or sets of actions intended to facilitate alignment between EHR and physician workflow from all relevant research. This encompasses observational studies, case studies, and any research that addresses related activities. Our primary objective is to understand how organizations have addressed the challenges of aligning physician workflows with EHR systems have been addressed. Also, this review will include studies related to electronic medical records. It does not consider the complexity of the information system’s network or interconnectivity, but rather focuses on the suitability of the information system with physician workflow. The studies may be based on the experience of a single physician or multiple physicians. Any additional features or interventions that do not address misalignment in the workflow will be excluded (Textbox 2). Similarly, studies that investigate the impact of EHR design on physician workflow or access physician workflow with a focus on meaningful use will also be excluded. The objectives of meaningful use are not centered around the alignment of EHR and physician workflow but rather on the impact of EHR on the quality of care and adoption.

#### Context

The study will involve physicians from diverse settings, not limited to hospitals and primary care, to gain insights into the strategies and tools they have adopted to align their workflows with the EHR. Although the workflow of physicians in primary health care, hospital settings, and specialized areas like surgery and pediatrics may vary, the study’s objective is to provide an understanding of the subject. Therefore, all studies conducted in hospital environments, primary health care, secondary, tertiary, and quaternary settings will be included. This includes studies from both the public and private sectors, as well as all areas of specialization such as emergency medicine, general practice, and surgery. There will be no bias toward specific countries or continents, except for articles not written in English (Textbox 2).

### Step 4: Charting and Synthesizing the Data

Data will be input into the Covidence systematic review software, developed by Veritas Health Innovations. This software will be used to remove duplicate articles, select relevant ones, and track the reviewers’ activities. Two independent reviewers will assess each article for eligibility during the screening process.

**Figure 1:**
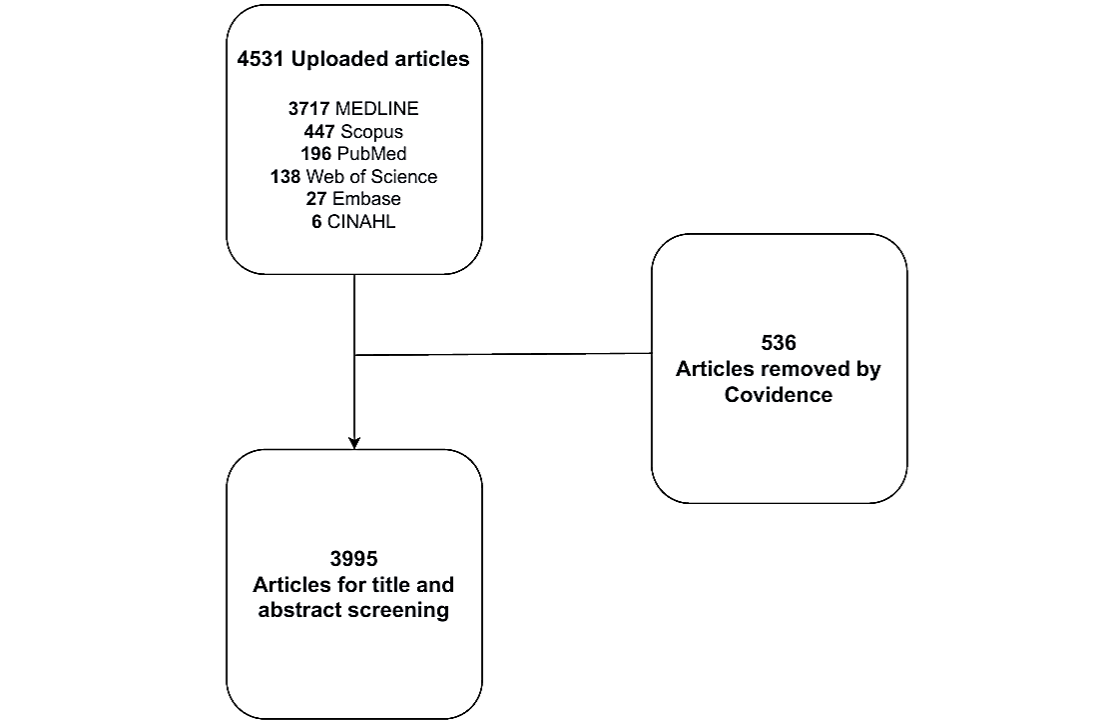
PRISMA (Preferred Reporting Items for Systematic Reviews and Meta-Analyses) flowchart.

The initial screening phase will involve 2 reviewers independently evaluating the publication’s title and abstract. However, if there is a disagreement, the final decision will be made by the third reviewer, who is a more experienced investigator (Figure 1).

Relevant variables will be extracted from selected studies during the full-text review. The articles will be extracted using the data extraction tool in the Covidence software. This tool will create a table using specified variables as its headers, allowing reviewers to complete the tables based on their findings. The extracted variables will cover the scope of the study on physician workflow and EHR alignment. Predetermined variables, such as health care setting, physician specialization, alignment challenges being addressed, and intervention or strategies (Textbox 3), will be extracted.

In the second phase of screening, the articles that pass the title and abstract screening will undergo a full article screening. Full articles will be assessed using the UWO online library. In cases where the article is not available at the UWO library, we will arrange for an interlibrary loan. In addition, if we are unable to access the article through interlibrary loan or if we require methodological details or clarification on missing or unclear data, we will contact the authors.

To ensure broader coverage, we will use the ancestry method, which involves screening the references of selected articles for relevance. Finally, we will use a PRISMA-ScR flow diagram to summarize the screening process, including the inclusion and exclusion criteria, number of included and excluded articles as well as the total number of articles selected for the review. Two reviewers will screen the full text of all articles selected during title and abstract screening to determine if they meet the eligibility criteria for extraction and where conflict arises, eligibility will be determined by the third reviewer who is a more experienced investigator.

**Variables that will guide data extraction.**

**Study design**

- Title
- Author
- Year of the study
- Country
- Aim of the study
- Data collection methods
- Setting: hospital, clinic, intensive care unit, internal medicine, and so on
- Physician specialization: primary care, pediatrics, surgery, and so on

**Study context and concept**

- Addressed challenges
- Intervention, strategy, or tool for alignment
- Impact or outcomes: challenges, advantages, and disadvantages

### Step 5: Collating, Summarizing, and Reporting the Results

The PRISMA-ScR extension flow diagram will be used to present the report of the scoping review [28]. The extracted data from the review will be qualitatively analyzed using frequency, common characterization of health care settings (level of care) or physician specialization, study design, and numerical descriptions such as the mean physician time taken to accomplish a task. Thematic analysis technique will be used to analyze the data. The reviewers will aim to identify common conceptual categories from the data, which will then be used to form themes relevant to answering the research questions. The results will be presented in a descriptive format that addresses the research questions by using an interpretive translation of the findings. Therefore, the report will consist of a narrative description of the findings.

## Results

We have completed a preliminary search of the identified databases and anticipate finishing the review process by July 2025. The extracted data will be presented in diagrammatic or tabular forms to align with the objective of this review. The report will include a narrative summary that accompanies the tabulated or charted results, in a way that relates to the review’s objective. Ultimately, these collated results and findings will be presented for peer-reviewed publication in a reputable journal. The result of this review will be published online and will be shared with relevant stakeholders, especially health care organizations and physicians.

## Discussion

### Anticipated Findings

The need to align EHR with physician workflow is critical for enhancing health care delivery and reducing physician burnout. Previous studies, such as those by Avendano et al [30] have explored specific EHR-physician alignment strategies, albeit in a limited scope by focusing on only 3 randomly selected strategies. Our scoping review aims to expand on this work by comprehensively identifying all strategies and tools used by organizations to facilitate the alignment of EHR systems with physician workflows.

In conducting preliminary searches in December 2024, we found no existing scoping review protocols or title registrations in the Cochrane and JBI databases that address this specific topic. This gap underscores the necessity of our review to synthesize the diverse strategies and tools used across different health care settings. The scoping review methodology is particularly suited for this endeavor, as it allows for the examination of a wide range of evidence, including diverse research innovations and their impacts. This approach will enable us to map the extent and nature of research activity in this area, identify knowledge gaps, and set the stage for future research agendas. By presenting a comprehensive overview of the strategies and tools used to align EHR with physician workflow, we will provide valuable insights for health care organizations aiming to optimize their EHR system. While utmost effort will be made to follow the guidelines of this protocol, any deviation, uncovered information, and identified limitations will be reported.

### Conclusions

This scoping review aims to offer a comprehensive synthesis of the strategies and tools that health care organizations use to align physician workflows with EHRs. By mapping current approaches, identifying gaps in the literature, and highlighting effective practices, this review will assist health care decision makers, inform the development of future interventions, and guide further research focused on improving EHR usability, reducing physician burnout, and enhancing patient care. The findings will be a valuable resource for stakeholders looking to optimize physician-EHR alignment across various clinical settings.

## Appendix

Multimedia Appendix 1 PRISMA-ScR checklist.

## Abbreviations

EHR: electronic health record
JBI: Joanna Briggs Institute
PCC: population, context, and concept
PRISMA-ScR: Preferred Reporting Items for Systematic Reviews and Meta-Analyses extension for Scoping Reviews
