# Supplementary material for: Strategies and Tools for Electronic Health Records and Physician Workflow Alignment: Protocol for a Scoping Review
Source: JMIR Res Protoc. 2025 Jun 2;14:e60464. doi: 10.2196/60464 (PMC12171643; doi:10.2196/60464)
Supplement: Multimedia Appendix 1 [file resprot_v14i1e60464_app1.docx]

**Preferred Reporting Items for Systematic reviews and Meta-Analyses extension for Scoping Reviews (PRISMA-ScR) Checklist**

| **SECTION** | **ITEM** | **PRISMA-ScR CHECKLIST ITEM** | **REPORTED ON PAGE #** |
| --- | --- | --- | --- |
| **TITLE** | | | |
| Title | 1 | Strategies and tools for EHR and physician workflow alignment: a scoping review protocol | 1 |
| **ABSTRACT** | | | |
| Structured summary | 2 | **Background**  Electronic health records (EHRs) have been widely adopted in healthcare systems globally, offering potential benefits in data accessibility, quality improvement, and enhanced patient outcomes. However, the alignment between EHRs and physician workflows remains a significant challenge, leading to negative impacts on physician well-being and patient care. While healthcare organizations have attempted various strategies to improve this alignment, critical gaps still persist, highlighting the need for a comprehensive understanding of existing approaches and their effectiveness as a means to chart effective strategies to align physician workflows with EHRs.  **Objective**  This scoping review aims to identify and synthesize the strategies and tools that healthcare organizations have employed to align physician workflows with EHRs, assess their impacts, and explore their strengths and limitations. This review will provide a toolkit for healthcare organizations and researchers, offering insights into effective alignment practices and identifying knowledge gaps for future research.  **Methods**  This scoping review will follow the JBI framework for scoping reviews, while incorporating the PRISMA-ScR checklist for transparent reporting. We will search multiple databases, including MEDLINE, PubMed, Cochrane, CINAHL, Scopus, EMBASE, and Web of Science, for relevant literature on tools, strategies, and interventions used to align physician workflows with EHRs. The review will focus on studies involving physicians in direct patient care across primary, secondary, tertiary, and quaternary care settings. Two independent reviewers will screen titles, abstracts, and full texts for inclusion. Data extraction will be performed using a standardized form, and findings will be narratively synthesized and presented in tables and charts.  **Results**  The study is expected to provide a comprehensive toolkit of strategies and tools for EHR-physician workflow alignment. This synthesis will offer healthcare organizations practical guidance for improving alignment and provide researchers with a foundation for identifying research gaps and future directions. The final report is planned for submission to an indexed journal in April 2024.  **Conclusion**  This scoping review will offer valuable insights into the strategies and tools implemented by healthcare organizations to align EHRs with physician workflows. By assessing the effectiveness and limitations of these approaches, the review aims to contribute to improved EHR usability, reduced physician burnout, and enhanced patient care. | 2 |
| **INTRODUCTION** | | | |
| Rationale | 3 | Despite the increasing number of studies on how to improve alignment between clinicians and EHR workflows, the existing knowledge is highly diverse and fragmented, creating gaps in our comprehensive understanding. Different studies focus on various aspects of EHR-physician workflow alignment, such as time management, stress levels, and patient outcomes. For example, some studies analyze the amount of time physicians dedicate to EHR tasks compared to patient interaction, while others assess the influence of EHR on physician productivity and job satisfaction. The studies in this field lack synthesis, as researchers often work in isolation instead of building upon each other's findings. Consequently, the body of knowledge remains fragmented, without a clear and unified direction for improvement. One of the purposes of a scoping review is to improve understanding about complex issues that affect healthcare workers and EHRs and to map existing tools to improve this challenge | 4 |
| Objectives | 4 | This study seeks to address the following key question: What strategies and tools do organizations use to align physician workflow with the EHR?  Population is Physicians, Concept; Concept Physician EHR workflow alignment or optimization; Context: Workflow of physicians who have direct contact with patients in any context (in-patient, out-patient, hospitals, and primary health care). This review is expected to meet the following objectives   1. To unveil the strategies and tools have healthcare organizations used to align physician workflows with EHR systems 2. To understand how these strategies and tools impacted the alignment between physician workflows and EHRs, healthcare delivery, and patient care? 3. To understand the strength and limitations of these strategies and tools? 4. To understand the gaps that currently exist in the literature and the opportunities that exist for future studies e | 5 |
| **METHODS** | | | |
| Protocol and registration | 5 | N/A | - |
| Eligibility criteria | 6 | Any article that focuses on the optimization of EHR for physician workflow. The study focus must be on physicians, EHR workflow optimization, and employing any strategy, tool, or intervention to achieve this objective. | 8 |
| Information sources | 7 | To answer the research questions, we will conduct a comprehensive search of several databases, including EMBASE (OVID), MEDLINE (OVID), PUBMED, COCHRANE CINHAL, and interdisciplinary databases such as WEB OF SCIENCE and SCOPUS. To ensure we capture all relevant articles, we will also screen the references of selected articles using the ancestry method. Our aim is to include scientific peer-reviewed studies and conference proceedings, encompassing all types of scientific study designs and approaches. Furthermore, we will conduct a thorough search for grey literature by identifying pertinent sources such as reports, conference proceedings, theses, and government documents. We will search databases such as ProQuest Dissertations and Theses and OpenGrey to access and hand pick studies that have not undergone peer review. Also, we will search conference proceedings for abstracts and presentations that may not have been published in journals and take time to explore the websites of healthcare organizations and government agencies. | 5, 6 |
| Search | 8 | Search: (#1) AND (#4)  ("physician workflow"[Title/Abstract] OR (("doctor s"[All Fields] OR "doctoral"[All Fields] OR "doctorally"[All Fields] OR "doctorate"[All Fields] OR "doctorates"[All Fields] OR "doctoring"[All Fields] OR "physicians"[MeSH Terms] OR "physicians"[All Fields] OR "doctor"[All Fields] OR "doctors"[All Fields]) AND "workflow"[Title/Abstract]) OR "clinician workflow"[Title/Abstract]) AND ("electronic health records"[MeSH Terms] OR "electronic health records"[MeSH Terms] OR "EHR"[Title/Abstract] OR "EMR"[Title/Abstract] OR "computerized provider order entry"[Title/Abstract] OR "electronic health records"[Title/Abstract]) | 6 |
| Selection of sources of evidence | 9 | Data will be uploaded into the Covidence systematic review software developed by Veritas Health Innovations. To ensure consistency in screening, a pre-screening activity where all five reviewers will first screen all publications to validate the inclusion and exclusion criteria will be conducted. Subsequently, reviewers will meet to discuss the results and amend the screening and data extraction manual before the actual screen. Two independent reviewers will screen each article for eligibility, with two reviewers screening each article based on the publication title and abstract based on the inclusion and exclusion criteria. A regular meeting will be scheduled to resolve disagreements between reviewers; however, when there is doubt, a more experienced investigator will resolve the issue.  A consensus report will be provided to the team to facilitate conversations on eligibility agreements. | 10 |
| Data charting process‡ | 10 | After the full-text review, data will be extracted into a standardized data extraction table designed in Microsoft Excel from the selected publications to be analyzed by two reviewers. Two reviewers will design the data extraction table by adding all the variables that need to be extracted from the publications. The Excel table will be standardized through the joint efforts of all five reviewers. The two reviewers will independently extract the characteristics of each selected article into a standardized extraction table and independently summarize their findings. These findings will be shared among the five reviewers, and a meeting will be held to further synthesize the findings. | 10 |
| Data items | 11 | The characteristics extracted from each article will be added to the following variables: author’s name, year of publication, setting, methodology, type of intervention, and outcome. Other variables will be determined as the study progresses. | 10, 11 |
| Critical appraisal of individual sources of evidence§ | 12 | N/A | N/A |
| Synthesis of results | 13 | A Microsoft Excel table will be standardized by all five reviewers, and two reviewers will independently extract data from the spreadsheet. Extracted data will be read by all reviewers, and meetings will be held to analyze the data using thematic analysis. Major themes will be generated and organized into study results. | 11, 12 |
| **RESULTS** | | | |
| Selection of sources of evidence | 14 | EMBASE (OVID), MEDLINE (OVID), PUBMED, COCHRANE CINHAL, and interdisciplinary databases such as WEB OF SCIENCE and SCOPUS. | 7 |
| Characteristics of sources of evidence | 15 | To be achieved when the review is actualized | N/A |
| Critical appraisal within sources of evidence | 16 | N/A | N/A |
| Results of individual sources of evidence | 17 | Results will be generated after the study is actualized | N/A |
| Synthesis of results | 18 | Results will be generated after the study ​​​​​​​is actualized. | N/A |
| **DISCUSSION** | | | |
| Summary of evidence | 19 | A summary will be provided when the review is actualized. | N/A |
| Limitations | 20 | While the utmost effort will be made to follow the guidelines of this protocol, any deviation, uncovered information, and identified limitations will be reported. | 10 |
| Conclusions | 21 | We will provide a toolkit that various organizations have used to address the misalignment between the EHR and physician workflow across various healthcare organizations and countries. ​​​​​​​Also, where possible, we will provide the impact of each tool and strategy. Thus, healthcare organizations can improve the alignment between implemented EHRs and physician workflow. It can help them identify pitfalls encountered in the past and avoid repeating these mistakes. | 10 |
| **FUNDING** | | | |
| Funding | 22 | This study is not funded. | 10 |
